# The cryo-EM structure of gastric H^+^,K^+^-ATPase with bound BYK99, a high-affinity member of K^+^-competitive, imidazo[1,2-*a*]pyridine inhibitors

**DOI:** 10.1038/s41598-017-06698-8

**Published:** 2017-07-26

**Authors:** Kazuhiro Abe, Jun Shimokawa, Mao Naito, Keith Munson, Olga Vagin, George Sachs, Hiroshi Suzuki, Kazutoshi Tani, Yoshinori Fujiyoshi

**Affiliations:** 1 Graduate School of Pharmaceutical Sciences, Nagoya University, Nagoya, 464-8601 Japan; 2 Cellular and Structural Physiology Institute, Nagoya University, Nagoya, 464-8601 Japan; 3 Core Research for Evolutional Science and Technology, Japan Science and Technology Corporation, Chiyoda, Tokyo 100-0004 Japan; 4 VA, GLAHS, Los Angeles, CA USA; 5 Laboratory of Molecular Electron Microscopy, Rockefeller University, New York, 10065 USA; 6 CeSPIA Inc., 2-1-1, Otemachi, Chiyoda, Tokyo, 100-0004 Japan

## Abstract

The gastric proton pump H^+^,K^+^-ATPase acidifies the gastric lumen, and thus its inhibitors, including the imidazo[1,2-*a*]pyridine class of K^+^-competitive acid blockers (P-CABs), have potential application as acid-suppressing drugs. We determined the electron crystallographic structure of H^+^,K^+^-ATPase at 6.5 Å resolution in the *E2*P state with bound BYK99, a potent P-CAB with a restricted ring structure. The BYK99 bound structure has an almost identical profile to that of a previously determined structure with bound SCH28080, the original P-CAB prototype, but is significantly different from the previously reported P-CAB-free form, illustrating a common conformational change is required for P-CAB binding. The shared conformational changes include a distinct movement of transmembrane helix 2 (M2), from its position in the previously reported P-CAB-free form, to a location proximal to the P-CAB binding site in the present BYK99-bound structure. Site-specific mutagenesis within M2 revealed that D137 and N138, which face the P-CAB binding site in our model, significantly affect the inhibition constant (*K*_i_) of P-CABs. We also found that A335 is likely to be near the bridging nitrogen at the restricted ring structure of the BYK99 inhibitor. These provide clues to elucidate the binding site parameters and mechanism of P-CAB inhibition of gastric acid secretion.

## Introduction

**Figure 1:**
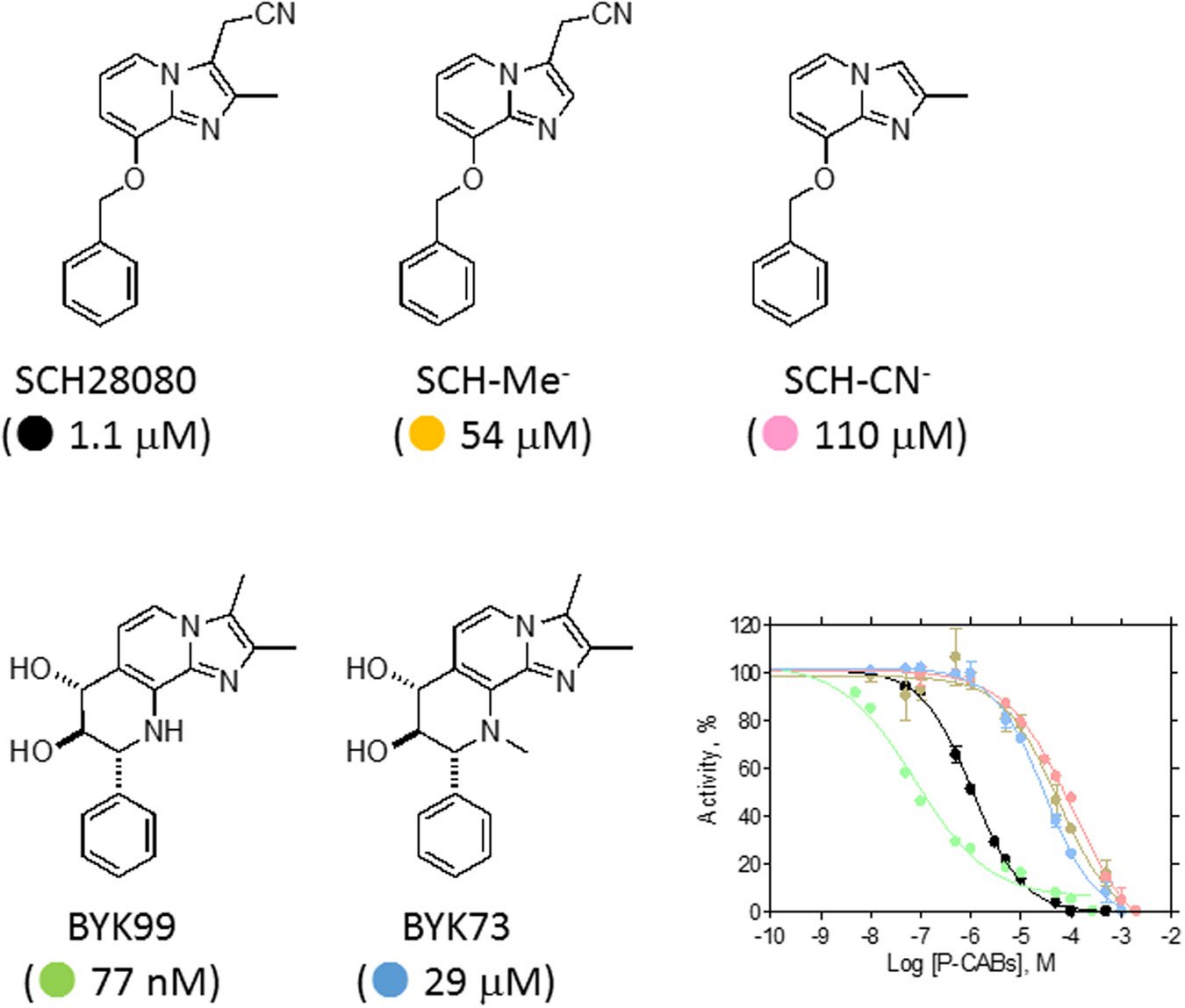
Chemical structures of SCH28080 and its derivatives. Chemical structures of SCH28080 and its derivatives used in this study are represented. Abbreviations for each compound are indicated. Dose-dependent inhibition of H^+^,K^+^-ATPase activity by these compounds in the presence of 10 mM KCl is shown in the graph. The half-inhibitory concentration of each compound at this K^+^ concentration (*IC*_*50*_) is indicated in parentheses.

The H^+^,K^+^-ATPase enzyme utilises ATP hydrolysis to drive electroneutral exchange of cytoplasmic protons for luminal K^+^, thus acidifying the gastric juice^1, 2^. Proton pump inhibitors (PPIs), such as omeprazole, are currently administered to suppress acid secretion in the treatment of acid-related diseases such as gastric ulcer or gastroesophageal reflux^3^. Acid suppression, in combination with antibiotics, is required for eradication of the gastric bacterium *Helicobacter pylori*. PPIs are weak bases that become protonated and accumulate in the acidic gastric lumen where they are converted to active sulfonamides and inhibit acid secretion by reacting covalently with cysteines accessible on the luminal side of H^+^,K^+^-ATPase (Fig. S1A)^4^. This mechanism results in a relatively slow onset of acid inhibition. The short dwell-times of PPIs in the blood and their instability under acidic conditions also negatively affect their efficacy as acid-suppressing drugs. Another class of H^+^,K^+^-ATPase drugs, K^+^-competitive acid blockers (P-CABs), are under development and some are now available for clinical use (Fig. S1B). The rapid and long-lasting acid suppression by P-CABs is expected to provide more immediate and efficient therapy for acid-related disease^5^. SCH28080 contains a imidazo[1,2-*a*]pyridine framework shared by a large subclass of P-CABs that competitively inhibit binding of the stimulating counterion, K^+^ (Fig. 1)^6^. Clinical application of SCH28080, however, is infeasible due to its toxicity. BYK99 has a chemical structure similar to that of SCH28080, except that the orientation of the imidazo[1,2-*a*]pyridine ring and the phenyl group is restricted by the addition of a diol bridge (Fig. 1), which results in BYK99 having more than 20-fold higher affinity than SCH28080^7^. Based on the mutually antagonistic binding of omeprazole and SCH28080^8^, as well as SCH28080 and BYK99^9^, we investigated the possible binding site overlap of these inhibitors. A detailed understanding of the SCH28080 and BYK99 binding sites could facilitate the rational design of improved acid-suppressing drugs.

We previously reported a cryo-electron microscopy (cryo-EM) structure for H^+^,K^+^-ATPase with bound SCH28080 at 7 Å resolution and identified its binding location in a cavity facing the gastric lumen^10^. We demonstrated that the SCH28080 binding site formed as a consequence of the conformational rearrangement of the enzyme structure that includes transmembrane helices M1 to M4 and the A domain with its connecting linker to the M2 helix^11, 12^. The EM density corresponding to the bound SCH28080 was only partially visible, however, and neither its orientation nor its detailed interactions with the surrounding amino acid residues could be determined explicitly. The present study demonstrates a different P-CAB, BYK99, binds in the same general cavity and generates a similar global binding conformation.

## Results and Discussion

### Analysis of the BYK99-bound structure

**Figure 2:**
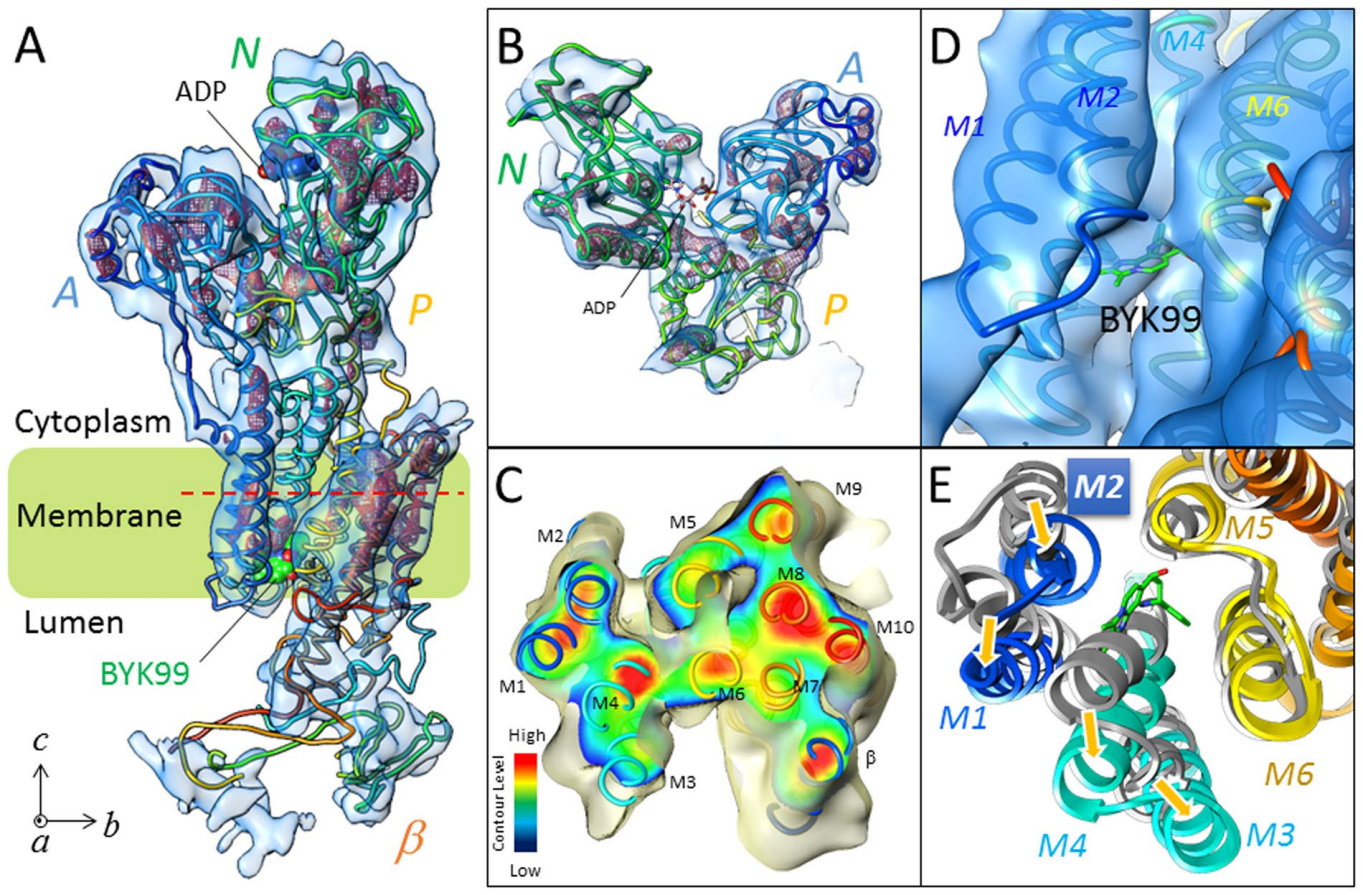
Electron crystallographic structure of BYK99-bound H^+^,K^+^-ATPase. (**A**) An EM map of the H^+^,K^+^-ATPase αβ complex in the BYK99-bound *E2*BeF state (blue surface: 1.5 σ, red mesh: 4 σ), with a superimposed homology model (ribbon, colour gradually changes from the N (blue) to the C (red) terminus of the α- and β- subunit). Several important structural components, cytoplasmic domains (A-, N- and P-domains), and bound ADP and BYK99 are highlighted in the figure. (**B**) Cytoplasmic domains viewed from the cytoplasmic side of the membrane. (**C**) Cross-section of the transmembrane helices at the indicated position (red dotted line in A), viewed from the luminal side perpendicular to the membrane normal. Surface colour shows the contour level at the indicated plane, the colour of which gradually changes from blue (low) to red (high) as indicated in the lower left. (**D**) BYK99-binding site viewed from luminal side of the membrane. Blue surface represents EM density with the 1 σ contour level. BYK99 (green stick) is manually superimposed on the EM density map to clarify its binding position. (**E**) Comparison of the arrangement of TM helices in the presence or absence of bound BYK99. A cross-section of the luminal TM region parallel to the membrane plane is viewed from the luminal side of the membrane. Homology models of the BYK99-bound form (colour ribbons as in A) and BYK99-free form (*E2*AlF, grey ribbons) are superimposed. Bound BYK99 (green sticks) would sterically overlap with the unbound protein conformation (grey). Yellow arrows indicate conformational rearrangement given by BYK99 binding from the structure without ligand. Transmembrane helix 2 (M2), evaluated in this study, is highlighted.

We employed electron crystallography of two-dimensional crystals to determine the structure of H^+^,K^+^-ATPase at 6.5 Å resolution. BYK99 and beryllium fluoride were added to generate the *E2*P state with bound BYK99 [(BYK)*E2*BeF] (Fig. 2, Table S1). The quality of the density map of the BYK99-bound form analysed at 6.5 Å resolution was significantly improved over that of the 7 Å structure with bound SCH28080 [(SCH)*E2*BeF]^10^. The density assigned to bound BKY99 was observed in the luminal opening (Fig. 2D, Fig. S2), in a position that well overlapped the previously identified SCH28080 binding site (Fig. S3BC). This finding is consistent with the previously reported mutually exclusive binding of the two inhibitors, SCH28080 and BYK99^9^. The molecular envelope of the cytoplasmic domains were well defined (Fig. 2B) and the transmembrane (TM) helices were observed as separate cylindrical densities in the (BYK)*E2*BeF structure (Fig. 2C, Fig. S2). These features are comparable to the other EM structures of H^+^,K^+^-ATPase so far determined. (BYK)*E2*BeF exhibited an almost identical molecular conformation as (SCH)*E2*BeF (Fig. S3A,D). Namely, the arrangement of the TM helices as well as the relative orientation of the cytoplasmic domains, and also the α-helical structure of the linker region connecting the A domain and M2 in the BYK99-bound form, were very similar to those of bound SCH28080^10^. When compared with the *E2*P transition state *E2*AlF^11^, where the luminal vestibule is closed, the (BYK)*E2*BeF (Fig. 2E) conformation exhibited an open vestibule where the M3M4 helix bundle bends outwardly at the luminal membrane interface and the M1-M2 helix bundle is near the bound BYK99. This conformation with an open vestibule is suggested to provide luminal access for the exchange of protons for K^+^ during the transport cycle^13^, and is similar to the ouabain-bound *E2*P state for Na^+^,K^+^-ATPase^13^ and the BeF-bound *E2*P “ground state” for SERCA^14^. The latter has also been proposed as the conformation associated with Ca^2+^ release to the lumen. These similarities among related P2-type ATPases suggest that the open *E2*P conformation is required for P-CABs to gain access to the binding cavity. Binding within the cavity would displace ordered water from both the cavity and the P-CAB, thus favouring the stability of the bound state. The present cryo-EM results provide direct evidence for a mechanism underlying the competitive inhibition by P-CABs, in which inhibitor binding blocks access of K^+^ from the lumen to its transport site and, conversely, binding of K^+^ at the transport site gives ion occlusion and closure of luminal access to the inhibitor binding cavity.

### Evaluation of the proximity of M2 and the P-CAB binding site by site-directed mutagenesis

Our structural analyses revealed overlapping binding positions for SCH28080 and BYK99 (Fig. S3) and a common molecular conformation for H^+^,K^+^-ATPase. The M2 helix was in close proximity to the P-CAB binding site in the P-CAB bound state, but largely displaced from this position in the absence of the inhibitor (Fig. 2E). Mutagenesis of M2 was therefore investigated to identify the side-chains important for high-affinity binding. The wild-type (WT) or mutant α-subunit of H^+^,K^+^-ATPase was co-expressed with the β-subunit in HEK293S GnT1^−^ cells^15^, and the ATPase activity in permeabilised membrane fractions was measured. In addition to SCH28080 and BYK99, we synthesised their derivatives: SCH-Me^−^, SCH-CN^−^ for SCH28080, and BYK73 for BYK99 (Fig. 1, Fig. S6), and characterised their affinities and modes of inhibition. Most of the mutants retained sufficient activity for characterisation of the K^+^-competitive kinetics of inhibition by SCH28080 (Table S2 and Fig. S4). Affinity (inhibition constant, *K*_i_) was calculated as described in the Materials and Methods^16^. Table 1 summarises the *K*_i_ of the compounds found for enzymes with a mutated M2. A striking finding was that mutations at amino acids D137 and N138 showed large changes in affinity for all compounds evaluated. The effects were similar in magnitude to some of those reported for mutations of amino acids likely to interact with bound SCH28080^17–23^, including A335C/C813A (*K*_i_ > 40,000 nM, ref. 20), L809F (*K*_i_ = 6150 nM)^20^, and Y799A (~100-fold low apparent affinity [*IC*_50_])^21^. Surprisingly, the N138A mutant exhibited more than 20-fold *higher* affinity for SCH28080 than for the WT enzyme. Similar increased affinity was observed for SCH-Me^−^ and BYK99, but not for SCH-CN^−^, suggesting that the smaller side-chain of the mutant relaxes the length or conformational constraints for inhibitor binding. In contrast, hydrophobic mutations N138L and N138I had differing effects on inhibitor affinity (Table 1), indicating that the hydrophobic surface in this part of the binding site is important. In marked contrast to the D137 and N138 mutants, mutations of other amino acids in the M2 helix (D136, L139, and Y140) exhibited affinities almost comparable to the WT enzyme for all evaluated compounds, clearly indicating that these amino acids are not directly involved in the binding of SCH28080 or other tested chemicals. Also, mutations of the M1 helix did not have a significant effect on P-CAB affinity (Table 2), indicating that M1 does not directly contribute to P-CAB binding. Mutagenesis of the M2 helix showed that only mutations at D137 and N138 affected SCH28080 affinity (Table 1). This part of M2 is in close proximity to the inhibitor after rearrangement of the TM helices to give the P-CAB bound structures (Fig. 2E); hence, the inhibitor binding site is bounded by M2 on one side, and by M4, M5, and M6 helices on the other.

**Table 1:** Effect of mutation in the M2 helix on H^+^,K^+^-ATPase.

position	mutation	*K*_*i*_[SCH28080]		*K*_*i*_[SCH-Me^−^]		*K*_*i*_[SCH-CN^−^]		*K*_*i*_[BYK99]		*K*_*i*_[BYK73]	
		nM	-fold^a^	μM	-fold	μM	-fold	nM	-fold	μM	-fold
WT	—	150 ± 10	1.0	11 ± 0.1	1.0	3.2 ± 0.3	1.0	5.8 ± 0.6	1.0	4.9 ± 0.4	1.0
D136	A	170 ± 20	1.1	11 ± 1	1.0	5.1 ± 0.4	1.6	10 ± 1	1.7	9.0 ± 0.5	1.8
	L	130 ± 10	0.9	5.4 ± 0.5	0.5	3.3 ± 0.2	1.0	5.3 ± 0.5	0.9	3.2 ± 0.2	0.7
	I	160 ± 20	1.1	6.8 ± 0.6	0.6	4.1 ± 0.3	1.3	15 ± 2	2.6	6.7 ± 0.4	1.4
	F	170 ± 20	1.1	6.7 ± 0.5	0.6	2.9 ± 0.1	0.9	5.9 ± 0.7	1.0	4.0 ± 0.2	0.8
D137	A	**2800** ± **200**	**10**	61 ± 7	5.5	28 ± 2	8.8	**81** ± **13**	**14**	**120** ± **30**	**25**
	L	**6000** ± **700**	**30**	**270** ± **100**	**25**	**32** ± **6**	**10**	**220** ± **40**	**38**	> **100**	> **20**
	I	**3200** ± **700**	**21**	**170** ± **40**	**16**	**39** ± **7**	**12**	**62** ± **15**	**11**	32 ± 5	6.5
	F	**2300** ± **400**	**13**	34 ± 7	3.1	**47** ± **6**	**15**	**640** ± **110**	**110**	**110** ± **10**	**22**
N138	A	*7*.*8* ± *0*.*1*	*0*.*05*	*0*.*56* ± *0*.*08*	*0*.*05*	4.2 ± 0.5	1.3	1.4 ± 0.3	0.2	6.6 ± 0.8	1.3
	L	840 ± 150	5.6	23 ± 7	2.1	14 ± 2	4.4	**1300** ± **500**	**220**	> **100**	> **20**
	I	53 ± 5	0.4	*0*.*95* ± *0*.*23*	*0*.*09*	3.7 ± 0.7	1.2	34 ± 8	5.9	**62** ± **17**	**13**
	F	**13000** ± **1000**	> **67**	**320** ± **300**	**29**	**52** ± **9**	**16**	**7700** ± **1200**	**1300**	> **100**	> **20**
L139	A	170 ± 10	1.1	8.7 ± 0.7	0.8	3.3 ± 0.3	1.0	14 ± 2	2.4	5.8 ± 0.6	1.2
	—										
	I	60 ± 5	0.4	2.3 ± 0.3	0.2	1.1 ± 0.1	0.3	2.6 ± 0.2	0.4	1.7 ± 0.2	0.3
	F	290 ± 20	1.9	13 ± 1	1.2	6.6 ± 0.5	2.1	18 ± 3	3.1	8.9 ± 0.7	1.8
Y140	A	210 ± 40	1.4	13 ± 1	1.2	7.3 ± 1.0	2.3	25 ± 3	4.3	14 ± 2	2.9
	L	140 ± 10	0.9	3.7 ± 0.4	0.3	2.7 ± 0.2	0.8	5.3 ± 0.4	0.9	6.5 ± 0.6	1.3
	I	120 ± 100	0.8	5.0 ± 0.8	0.5	2.1 ± 0.2	0.7	4.3 ± 0.5	0.7	4.0 ± 0.5	0.8
	F	360 ± 40	2.4	21 ± 3	1.9	3.7 ± 0.2	1.2	35 ± 6	6.0	7.6 ± 0.5	1.6
L141	A	*n*.*d*.^b^	—	*n*.*d*.	—	*n*.*d*.	—	*n*.*d*.	—	*n*.*d*.	—
	—										
	I	300 ± 60	2.0	7.3 ± 0.8	0.7	2.4 ± 0.2	0.8	8.4 ± 1.5	1.4	3.9 ± 0.8	0.8
	F	*n*.*d*.	—	*n*.*d*.	—	*n*.*d*.	—	*n*.*d*.	—	*n*.*d*.	—

The inhibition constant (*K*_*i*_) for the indicated compounds (Fig. 1) determined by K^+^-competitive inhibition of H^+^,K^+^-ATPase activity (Fig. S4) is shown. Data represent the mean ± SEM determined by fitting 96 data points for each experiment. ^a^Value indicates x–fold increase in *K*_*i*_ value of each mutant compared with that of wild-type. ^b^Not determined due to its low ATPase activity. *K*_*i*_ values largely affected by mutagenesis are highlighted (Bold and underline: > 20-fold higher *K*_*i*_, Bold: 20-fold > *K*_*i*_ > 10-fold, Italic: < 1/20 fold lower *K*_*i*_).

**Table 2:** Effect of mutation in the M1 helix on H^+^,K^+^-ATPase.

TM	mutant	*K*_*i*_[SCH28080]		*K*_*i*_[BYK99]	
		nM	-fold^a^	nM	-fold
—	WT	150 ± 10	1.0	5.8 ± 0.1	1.0
M1	A125S	440 ± 20	2.9	27 ± 2	4.6
	A125F	180 ± 10	1.2	12 ± 1	2.1
	I126A*	1100 ± 100	7.3	**110** ± **50**	**19**
	I126F*	590 ± 60	3.9	24 ± 15	4.1
	Q127A	210 ± 20	1.4	19 ± 1	3.3
	Q127F	450 ± 50	3.0	41 ± 7	7.1

Data are shown as in Table 1. Asterisks indicate mutants exhibiting high H^+^-ATPase activity, whose *K*_*i*_ was determined by a *K*_m_/*V*_max_ plot (see Materials and Methods for details).

### Effect of negatively-charged residue at the M2 helix

SCH28080 and other P-CABs behave as weak bases with the p*Ka* of imidazo[1,2-*a*]pyridine (p*Ka* = 5.6) giving only approximately 2.5% protonation at the pH of our ATPase assays (pH 7.0). It was previously proposed, however, that protonated SCH28080 binds with higher affinity. D137 is the only acidic amino acid in the M2 helix near the binding site in our model, and its accessibility to the solvent in the absence of the inhibitor is expected to give it a negative charge. This led us to evaluate a possible charge effect at the position of D137 (Table 3). As shown in Table 3, a D137N mutant displayed more than 60-fold lower affinity for both SCH28080 and BYK99 (*K*_i_ > 10,000 nM and 450 nM, respectively) than WT. The magnitude of these differences suggests that strong charge-pairing between D137 and the imidazo[1,2-*a*]pyridine ring is important for the binding. Although the *K*_i_ value of the charge-conserved mutant D137E could not be readily determined from global fitting due to its unusual kinetic parameters (see Materials and Methods), its affinity (*K*_i_’) determined from a *K*_m_/*V*_max_ plot indicated a smaller effect on the SCH28080 and BYK99 binding affinity (*K*_i_’[SCH28080]D137E = 170 nM and *K*_i_ [BYK99]D137E = 57 nM, respectively). Introduction of a negative charge at the neighbouring N138 (N138D) had no significant effect on P-CAB affinity. Although the charge-swapped double mutant (D137N/N138D) exhibited reduced affinities for the P-CABs compared with the WT, its effect was much smaller than that of the single D137N mutant. This observation indicates that introducing a negative charge at the position close to D137 can partially, but not completely, compensate for the charge-neutralisation in the D137N mutant, and thus the correct orientation of the charged side-chain is also important for inhibitor binding. Therefore, the negatively charged side-chain of D137 in the M2 helix is in proximity to the imidazo[1,2-*a*]pyridine of SCH28080 and other related P-CABs, perhaps directly stabilising the positively-charged inhibitor where tight binding in the site would exclude water and prevent access to the bulk solvent. Under physiological conditions where the luminal pH is low, exclusion of water in the bound state could also prevent protonation of D137 to preserve the charge interaction. These data however could not exclude the possibility that the negatively-charged D137 indirectly affects inhibitor affinity. For example, D137 may be important for formation of the binding site itself by interacting with neighbouring side-chains.

**Table 3:** Effect of mutation in D137, N138, and previously reported residues of H^+^,K^+^-ATPase.

TM	mutation	*K*_*i*_[SCH28080]		*K*_*i*_[SCH-Me^−^]		*K*_*i*_[SCH-CN^−^]		*K*_*i*_[BYK99]		*K*_*i*_[BYK73]	
		nM	-fold^a^	μM	-fold	μM	-fold	nM	-fold	μM	-fold
WT	WT	150 ± 10	1.0	11 ± 0.1	1.0	3.2 ± 0.3	1.0	5.8 ± 0.6	1.0	4.9 ± 0.4	1.0
M2	D137E*	170 ± 100	1.1	*n*.*d*.	—	4.4 ± 0.4	1.4	57 ± 16	9.8	*n*.*d*.	—
	D137N	> **10000**	> **67**	*n*.*d*.	—	**120** ± **30**	**38**	**450** ± **100**	**78**	*n*.*d*.	—
	N138D	200 ± 20	1.3	*n*.*d*.	—	2.6 ± 0.2	0.8	7.7 ± 0.7	1.3	*n*.*d*.	—
	D137N/N138D	**1800** ± **200**	**12**	*n*.*d*.	—	23 ± 20	7.2	**110** ± **10**	**19**	*n*.*d*.	—
M4	A335G	60 ± 5	0.4	1.6 ± 0.2	0.15	1.0 ± 0.1	0.3	1.2 ± 0.1	0.2	*0*.*078* ± *0*.*006*	*0*.*016*
	A335V	> **10000**	> **67**	*n*.*d*.	—	*n*.*d*.	—	> **1000**	> **170**	*n*.*d*.	—
	A335C/C813A	> **10000**	**>67**	**4200** ± **2300**	**382**	**71** ± **10**	**22**	> **1000**	> **170**	*n*.*d*.	—
M5	Y799A	> **10000**	> **67**	**740** ± **170**	**67**	**81** ± **15**	**25**	> **1000**	> **170**	> **200**	> **40**
	Y799F	880 ± 60	5.9	31 ± 3	2.8	18 ± 2	5.6	**110** ± **10**	**19**	21 ± 2.7	4.3
M6	L809F	**4400** ± **800**	**29**	**110** ± **20**	**10**	**98** ± **12**	**31**	**850** ± **130**	**150**	*n*.*d*.	—

Data are shown as in Tables 1 and 2.

### Mutants of the M3-M6 helices

Previous extensive mutagenesis studies suggested some key amino acids likely to interact directly with SCH28080^17–23^. To confirm that our measurements were consistent with these results, and to confirm whether low-affinity SCH28080 derivatives (SCH-Me^−^, SCH-CN^−^, and BYK73) show binding behaviour similar to the parent compound, we selected various previously reported mutations and assayed them in the presence of these compounds (Table 3). C813 (M6) forms a covalent S-S bond with omeprazole, and omeprazole binding is antagonistic with respect to SCH28080^3^. We therefore tested the double mutant A335C (M4)/C813A (M6), which has almost no affinity for SCH28080^20^. Y799A (M5) has a 100-fold reduced apparent affinity (*IC*_50_) for SCH28080, while the Y799F mutant has negligible effects on SCH28080 affinity, suggesting the importance of the phenyl group on this tyrosine residue^21^. Mutation of L809 for phenylalanine (M5M6 loop) led to a more than 90-fold reduced affinity. These mutations all showed similar results when reproduced in the present study, and the derivatives shown in Fig. 1 and Table 3 exhibited similar tendencies in their affinity changes. In addition, all of the compounds evaluated in this study showed competitive inhibition with respect to the K^+^ concentration regardless of their different affinities (Fig. S5). Therefore, we concluded that, despite the significantly lower affinity of SCH-Me^−^, SCH-CN^−^, and BYK73 compared with the original compounds, these derivatives apparently share a similar binding mode.

A remarkable difference was observed between the WT and A335G mutant with respect to their affinities for BYK73 (Table 3), a compound in which the bridging nitrogen is methylated (Fig. 1). Due to this methyl group modification, the WT enzyme has a 1000-fold lower affinity for BYK73 compared with BYK99 (*K*_i_[BYK73]WT = 4.9 μM and *K*_i_[BYK99]WT = 5.8 nM). Substitution of A335 with the smaller glycine partially compensates for the effect of the methyl group with more than 60-fold increased affinity for BYK73 (*K*_i_[BYK73]A335G = 78 nM). This contrasts with the 5-fold increase for BYK99 (*K*_i_[BYK99]A335G = 1.2 nM), suggesting that the secondary amine at the restricted ring structure of BYK99 binds in a narrow region next to A335^10, 24^. The A335G mutation apparently provides a better fit in the binding site and can partially compensate for the increased volume of the additional methyl group in BYK73. This notion is also supported by the A335V mutant, which has largely reduced affinities for BYK99 (*K*_i_[BYK99]A335V > 1000 nM) and SCH28080 (*K*_i_[SCH28080]A335V > 10,000 nM). It should be noted, however, that these results do not necessarily require that the methyl group of bound BYK73 replaces the side-chain methyl of A335 in the A335G mutant.

### Proposed model of P-CAB binding pocket on H^+^,K^+^-ATPase

**Figure 3:**
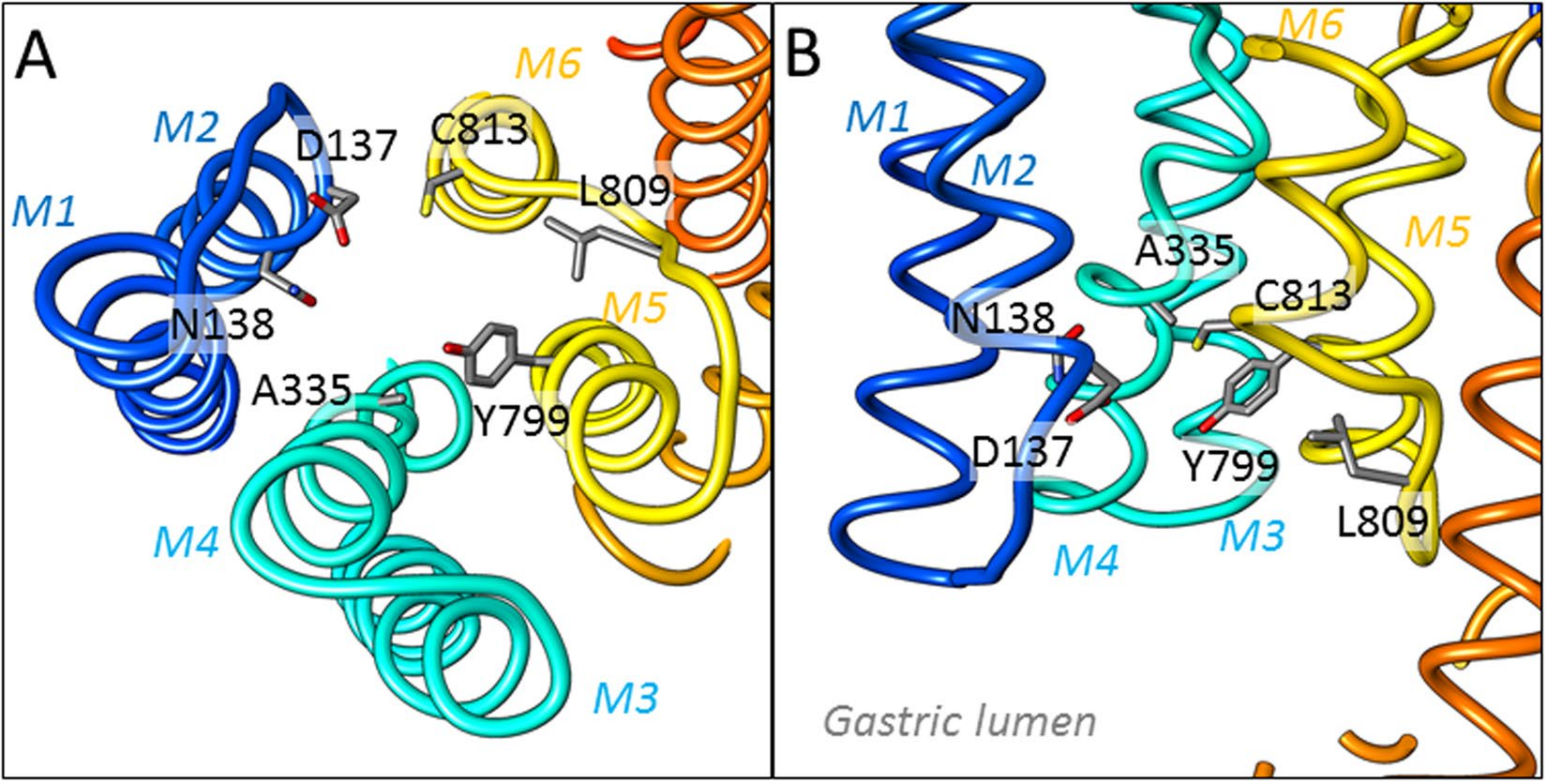
Model of the P-CAB binding site on H^+^,K^+^-ATPase. Closed view of the luminal cavity region in the homology model of H^+^,K^+^-ATPase (ribbons, colours are as in Fig. 2), viewed from luminal side of the membrane (**A**) and parallel to the membrane normal (**B**). Amino acids important for P-CAB binding determined by present and previous mutagenesis studies are shown as stick representations.

Based on the present as well as previous results, we suggest a model for the P-CAB binding pocket of H^+^,K^+^-ATPase (Fig. 3). From EM analysis and homology modelling, we were able to define the molecular conformation of gastric H^+^,K^+^-ATPase and the binding position of the P-CAB BYK99 within the luminal-facing cavity. Based on our mutagenesis data, amino acids contributing to the P-CAB binding site were mapped in the model. These amino acids were distributed in M2, M4, M5, and M6, and surrounded the cavity facing the gastric lumen, which is consistent with the proposed P-CAB binding position (Fig. 2D,E). The location of the binding site was also consistent with mutants that do not affect P-CAB affinity (Table S3). Our cryo-EM analysis and mutagenesis data suggest that the imidazo[1,2-*a*]pyridine ring aligns between the M2 helix (Table 1) on one side and A335 in the M4 helix on the other side. A similar location for the imidazo[1,2-*a*]pyridine class of inhibitors, such as SCH28080 and BYK99, was proposed in previous studies^24^ that also fits the cryo-EM density presented here. In that proposed model, the imidazo[1,2-*a*]pyridine ring is fitted similarly in the luminal vestibule, but the *para* position of the phenyl group faces toward TM1, thus supporting the known mutational effects on binding as well as the structure-activity relationships given by synthetic modification of the imidazo[1,2-*a*]pyridine ring^25^, and the photoaffinity labelling within the TM1/TM2 segment of H^+^,K^+^-ATPase given by a radioactive *para*-azidophenyl analogue of SCH28080^26^. Future studies with higher-resolution cryo-EM or X-ray crystallographic analysis is required to specify the detailed molecular orientation of the P-CABs for discrimination of these binding site models.

## Methods

### Electron crystallographic analysis

Two-dimensional crystallization and structural analysis of the BYK99-bound *E2*BeF structure by electron crystallography were performed as described previously^10^. Briefly, a purified membrane fraction from pig stomach^27^ was solubilised with octaethylene glycol monododecyl ether, mixed with dioleoyl phosphatidylcholine, and dialysed against buffer containing 10 mM propionate, 10% glycerol, 1 mM BeSO_4_, 4 mM NaF, 100 μM BYK99, and 3 mM dithiothreitol at 3 °C for ~2 weeks. The carbon sandwich method^28^ was applied for the cryo grid preparation and data were collected by cryo-EM with a liquid helium-cooled stage operated by 300 keV (JEM-SF3000F)^29^. Micrographs were analysed using MRC image processing programs^30–32^.

### Homology modelling

Homology models of the SCH28080- or BYK99-bound H^+^,K^+^-ATPase in the *E2*BeF state were built with MODELLER v9.7^33^, using the atomic model of ouabain-bound Na^+^,K^+^-ATPase (PDB ID: 4HYT)^28^ as a starting template. The initial manual fitting of the homology model into the density map was followed by adjustments for each individual domain and the TM helices within the EM density map using SITUS^34^. After the positional search, further fine fitting and connecting the split loop region was performed manually using COOT^35^ with regularisation refinement. Figures were prepared using UCSF Chimera^36^.

### Chemical synthesis of SCH28080 derivatives

SCH28080 and its derivatives were synthesised according to Kaminski *et al*.^25^.

### Expression of recombinant H^+^,K^+^-ATPase

The cDNA encoding the pig WT or mutant H^+^,K^+^-ATPase α-subunit and WT β-subunit were independently cloned into the BacMam vector^37^. The pig H^+^,K^+^-ATPase was expressed using baculovirus-mediated transduction of mammalian HEK293S GnT1^-^ cells, as described previously^15^. Membrane fractions were prepared by breaking cells with a Teflon homogeniser as described previously^19^ and used for the following ATPase assay.

### Determination of *K*_*i*_ for SCH28080 by 96-well format ATPase assay

Before measuring the ATPase activity, the membrane fraction (4 mg/ml) was permeabilised by incubating 10 mM HEPES/Tris, pH 7.0, 10% glycerol, and 0.1% β-Escin for 10 min at room temperature to make substrates and inhibitors fully accessible, and then diluted 10 times on ice with 250 mM sucrose and 0.5 mM EGTA/Tris, pH 7.6, to stabilise the enzyme for a minimum of one day, based on the H^+^,K^+^-ATPase activity^38^. Permeabilised membrane fractions (~2 μg total protein/80 μl) were suspended on ice in buffer comprising 40 mM PIPES, pH 7.0, 2 mM MgCl_2_, 2 mM ATP, and 0–30 mM KCl in the presence or absence of the indicated concentrations of SCH28080, BYK99, or other derivatives in the 96-well plate. Reactions were initiated by incubating at 37 °C using a thermal cycler, and maintained for 1 to 5 h (depending on the activity). Reactions were terminated by withdrawing 40 μL from the 80 μL reactant, and mixing with 80 μL of stop solution comprising 6% ascorbic acid and 2% ammonium molybdate in 1 N HCl, followed by the addition of 120 μL of 2% sodium arsenate, 2% sodium citrate, and 2% acetic acid. The amount of released inorganic phosphate was determined colorimetrically^39^ from absorbance at 850 nm by the microplate reader (TECAN).

Data measured from the 96-well plate contained triplicates of four different sets of K^+^-dependent ATPase assays in the absence or presence of three different concentrations of inhibitor to give 96 data points. Data were corrected for background values in the absence of K^+^ at each inhibitor concentration and fit by using simultaneous nonlinear regression (global fitting)^16^

$$v=\frac{{V}_{max}\cdot [S]}{{K}_{m}(1+\frac{[I]}{{K}_{i}})+[S]}$$

where: *v* is the ATPase activity, *V*_max_ is the maximal ATPase activity, *K*_m_ is the Michaelis constant for K^+^, *K*_i_ is the inhibitor constant, [S] is the K^+^ concentration, and [I] is the inhibitor concentration. Data were fitted by GraphPad Prism to obtain the mean and SEM.

Some of the mutants exhibited unusually high H^+^-ATPase activity, which is the reaction rate responsible for the spontaneous *E2*P dephosphorylation in the absence of K^+^ (*i*.*e*., basal activity). In the case of WT or most of the mutants, H^+^-ATPase activity was less than 5% of the total H^+^,K^+^-ATPase activity, having only a negligible effect on the determination of *K*_i_. In some mutants, however, significant amounts of H^+^-ATPase (>20% of the total H^+^,K^+^-ATPase activity) led to an underestimation of *V*_max_ value in the absence of P-CABs. For this reason, the *K*_i_ value of the mutants with high H^+^-ATPase activity could not be determined by global fitting. In these cases, we determined the *K*_i_ value (defined in this manuscript as *K*_i_’), from the *K*_*m*_/*V*_*max*_ plot^19^. These two procedures for the determination of *K*_i_ are essentially the same^16^, as we confirmed for the WT enzyme with SCH28080 (*K*_i_ (global fitting) = 150 nM, *K*_i_’ (*K*_*m*_/*V*_*max*_ plot) = 110 nM).

### Data Availability

The EM density map has been deposited in the EMDataBank, http://www.emdatabank.org/ [accession code EMD-6799]. The homology model of (BYK)E2BeF has been deposited in the Protein Data Bank, http://www.pdb.org (PDB ID code 5Y0B).

## Electronic supplementary material

Supplementary information
