# Supplementary material for: The cryo-EM structure of gastric H+,K+-ATPase with bound BYK99, a high-affinity member of K+-competitive, imidazo[1,2-a]pyridine inhibitors
Source: Sci Rep. 2017 Jul 26;7:6632. doi: 10.1038/s41598-017-06698-8 (PMC5529566; doi:10.1038/s41598-017-06698-8)
Supplement: Supplementary file 1 — Supplementary information [file 41598_2017_6698_MOESM1_ESM.pdf]

Supplementary Information

The cryo-EM structure of gastric H^+,K^+-ATPase with bound BYK99, a high-affinity member of K^+-competitive, [1,2-*a*] imidazopyridine inhibitors

Kazuhiro Abe[1,2,3,*], Jun Shimokawa[1], Mao Naito[1,2], Keith Munson[4], Olga Vagin[4], George Sachs[4], Hiroshi Suzuki[5], Kazutoshi Tani[2] & Yoshinori Fujiyoshi[2,3,6]

[1]Graduate School of Pharmaceutical Sciences and [2]Cellular and Structural Physiology Institute, Nagoya University, Nagoya 464-8601, Japan; [3]Core Research for Evolutional Science and Technology, Japan Science and Technology Corporation, [4]VA, GLAHS, Los Angeles, CA, USA, [5]Laboratory of Molecular Electron Microscopy, Rockefeller University, [6]CeSPIA Inc., 2-1-1, Otemachi, Chiyoda, Tokyo, 100-0004, Japan

[*]To whom correspondence should be addressed. E-mail: kabe@cespi.nagoya-u.ac.jp.

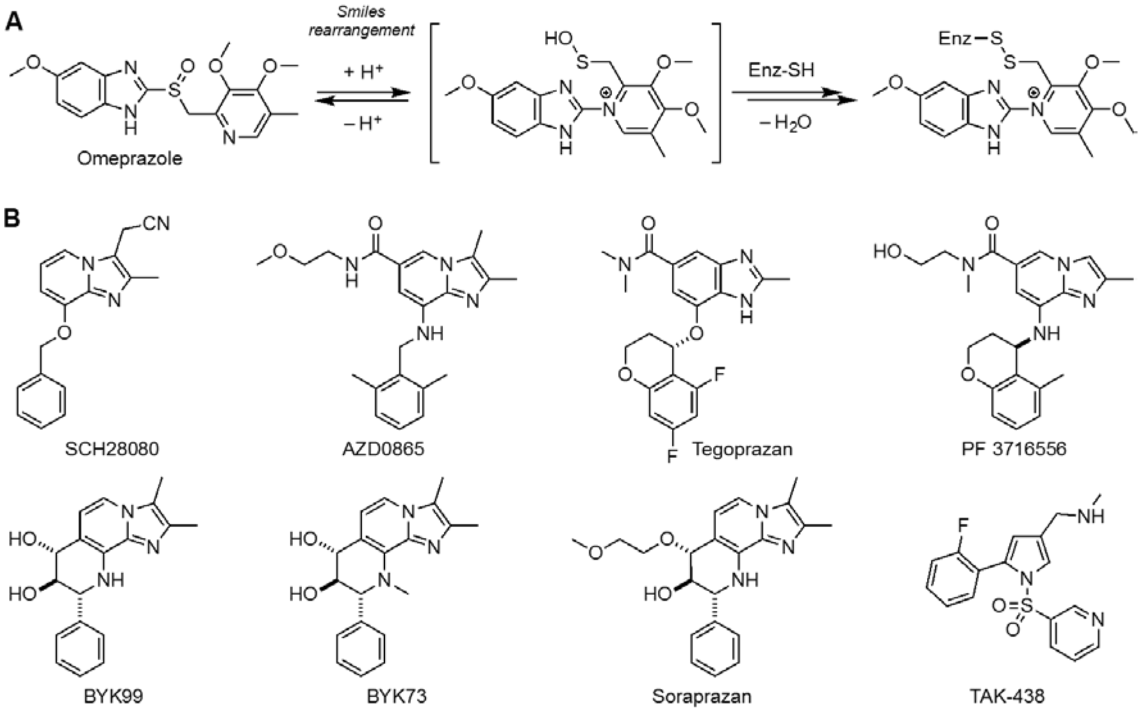

Fig. S1 Chemical structures of reported omeprazole (PPI) and P-CABs.

(A) A representative PPI, omeprazole, is converted to active sulfonamides in the acidic stomach, and reacts covalently with conserved C813 accessible from the luminal side of the H^+,K^+-ATPase (indicated as Enz-SH). (B) Chemical structure of P-CABs.

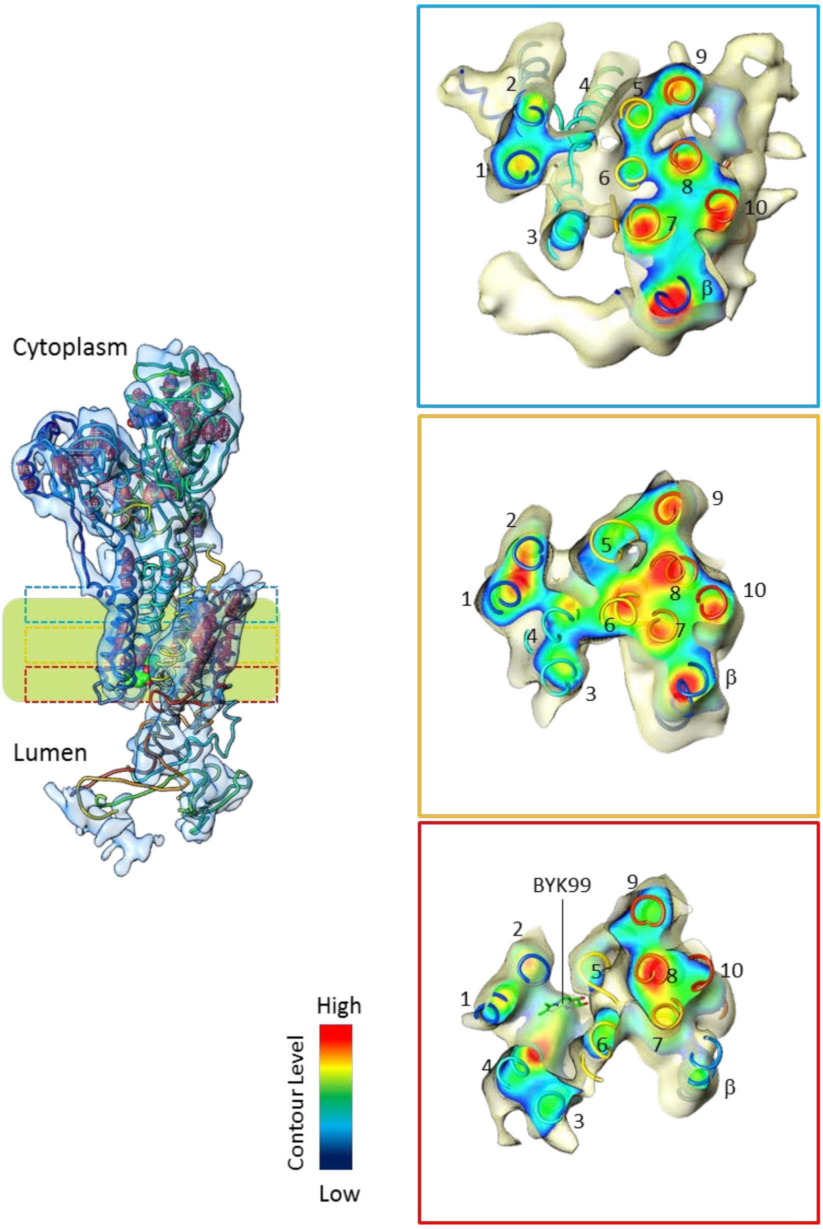

Fig. S2 Transmembrane structure showing slice representations of BYK99-bound H^+,K^+-ATPase at 6.5Å resolution

TM slices (right panels) at indicated positions in the whole structure (left), viewed from the luminal side of the membrane. Colour codes for EM map, homology models (ribbon), and sliced surface are as in Fig. 2.

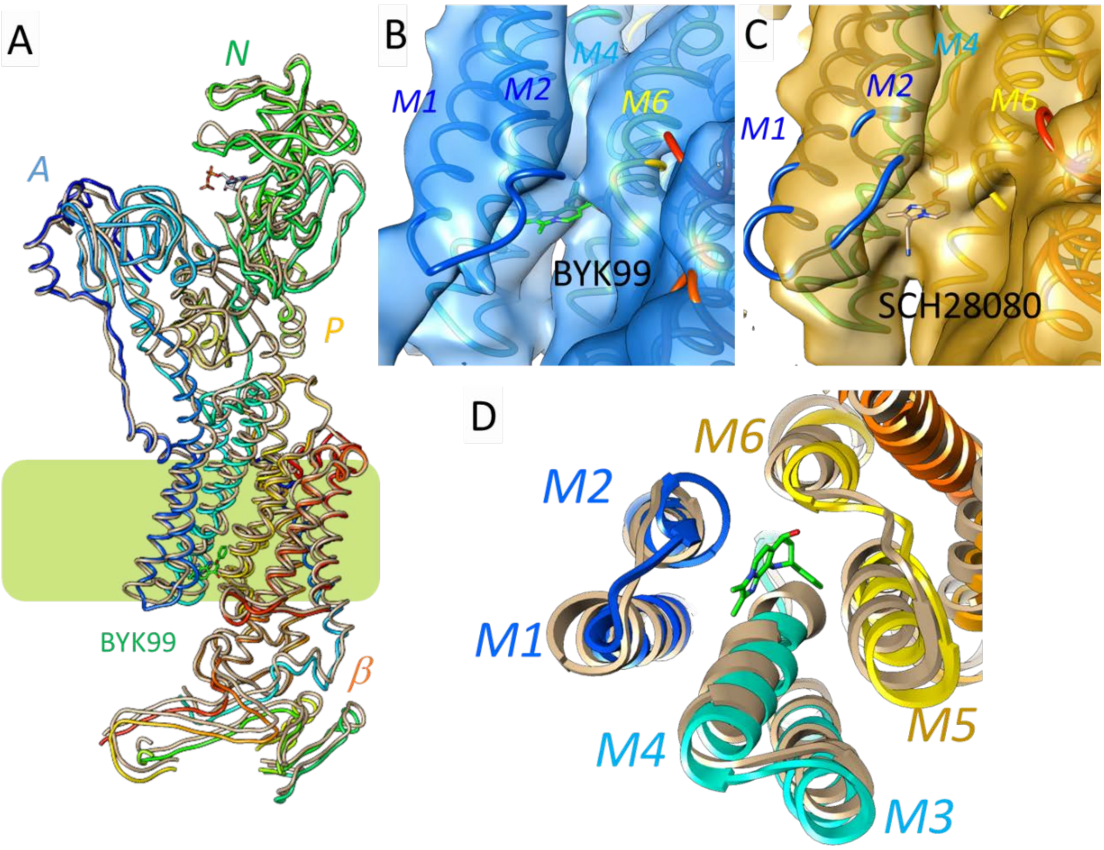

Fig. S3 Identical molecular conformations of SCH28080- and BYK99-bound H^+-K^+-ATPase. (A) Superimposition of homology models of SCH28080-bound (wheat ribbons, (SCH)*E2*BeF) and BYK99-bound form (colour ribbons, (BYK)*E2*BeF). (B,C) Comparison of the P-CAB binding site in the BYK99-bound (B) and SCH28080-bound forms (C). Each surface shows an EM map with contour level of 1σ. (D) Comparison of the helix arrangement in SCH28080- (wheat ribbons) and BYK99-bound forms (colour ribbons with bound BYK99 in green stick) revealed an almost identical molecular conformation in the TM region.

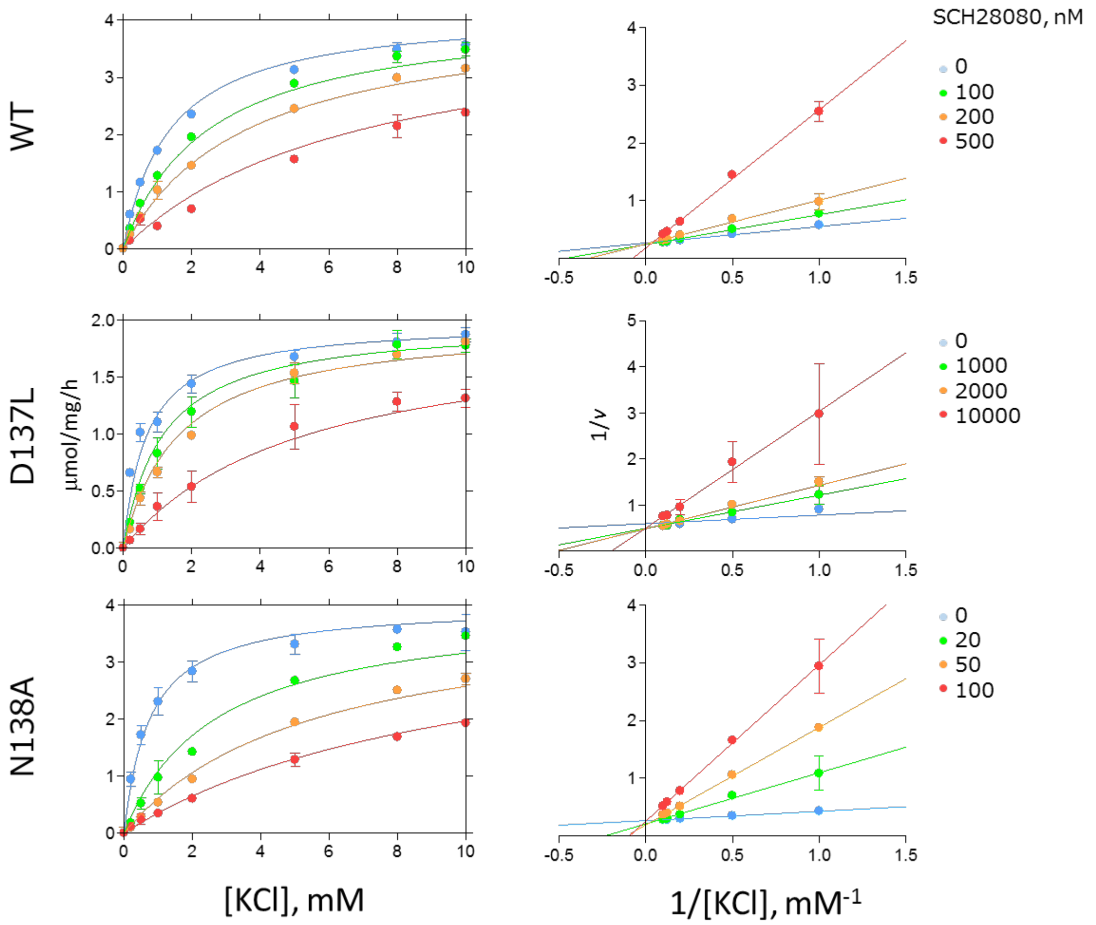

Fig S4 K^+-competitive inhibition of H^+,K^+-ATPase activity by SCH28080.

Michaelis-Menten plot (left row) and the inverse plot of 1/*v* vs 1/[K^+] (right row) for wild-type (WT) (upper line), D137L (middle line), and N138A (lower line) under different concentrations of SCH28080 (indicated in the figure with different colours). The stepwise increase of the K_m in the presence of increasing SCH28080 concentrations with constant V_{max} indicates that SCH28080 inhibition remains strictly competitive with K^+. Data were globally fit by simultaneous nonlinear regression (colour lines in the left row) to determine the inhibition constant (K_i) (see Materials and Methods). In the inverse plots, data were fit by linear regression (colour lines in the right row), showing the overlapped intercept on *y*-axis typical for competitive inhibition kinetics. Data plotted represent mean ± SEM of triplicate points, in eight K^+ concentrations and four SCH28080 conditions in single 96-well format

ATPase measurement for each WT and mutant enzyme.

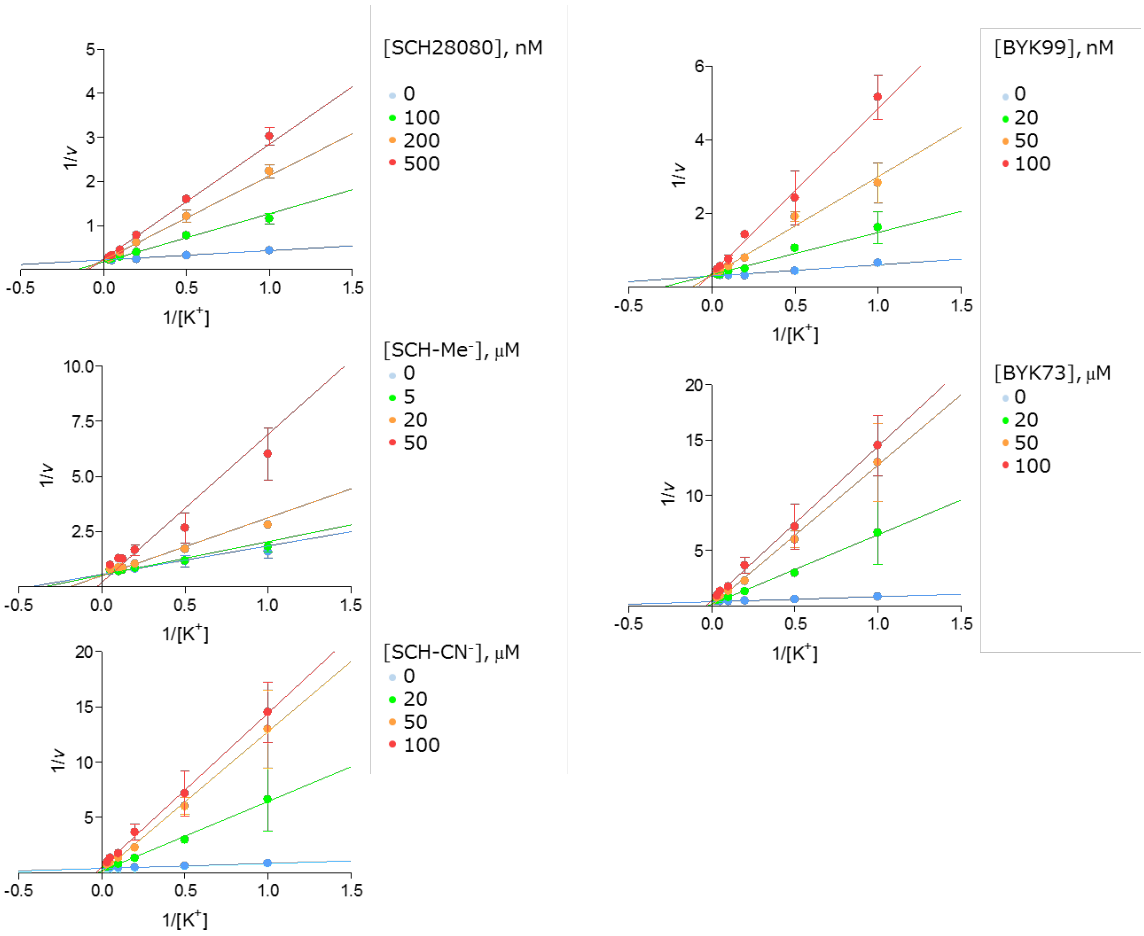

Fig. S5 K^+-competitive nature of evaluated P-CABs and its derivatives

The inverse plots for wild-type H^+,K^+-ATPase activity in the presence of different concentrations of the evaluated compounds, showing their competitive nature regardless of their affinity.

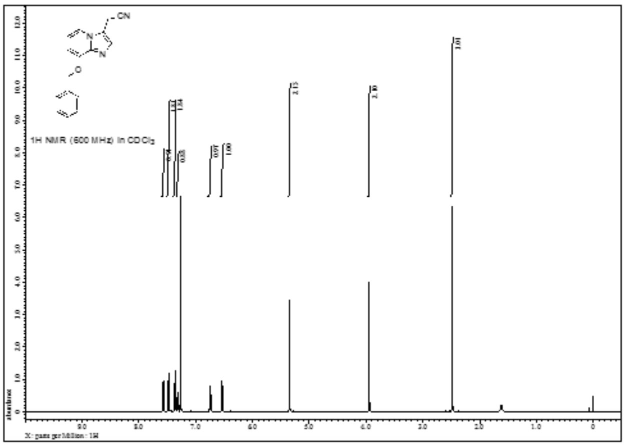

SCH28080

^{1}H NMR ($CDCl_3$, 600 MHz) δ
7.57 (d, *J* = 6.9 Hz, 1H),
7.48 (d, *J* = 7.6 Hz, 2H),
7.36 (t, *J* = 7.6 Hz, 2H),
7.31 (t, *J* = 7.6 Hz, 1H),
6.74 (dd, *J* = 7.6, 6.9 Hz, 1H),
6.53 (d, *J* = 7.6 Hz, 1H),
5.35 (s, 2H),
3.94 (s, 2H),
2.49 (s, 3H).

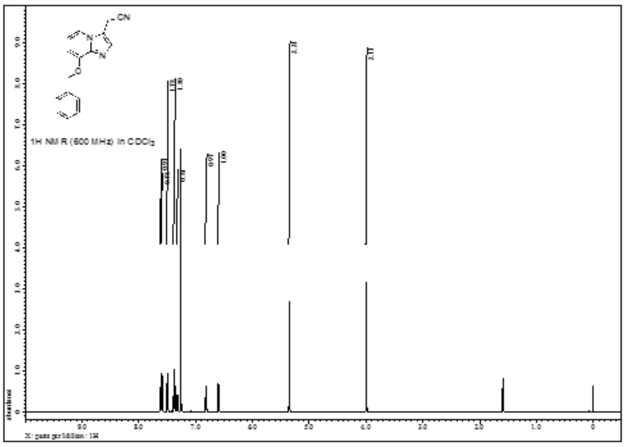

SCH-Me^{-}

^{1}H NMR ($CDCl_3$, 600 MHz) δ
7.62 (d, *J* = 6.9 Hz, 1H),
7.59 (s, 1H),
7.50 (d, *J* = 7.6 Hz, 2H),
7.38 (t, *J* = 7.6 Hz, 2H),
7.32 (t, *J* = 7.6 Hz, 1H),
6.82 (dd, *J* = 7.6, 6.9 Hz, 1H),
6.60 (d, *J* = 7.6 Hz, 1H),
5.35 (s, 2H),
3.99 (s, 2H).

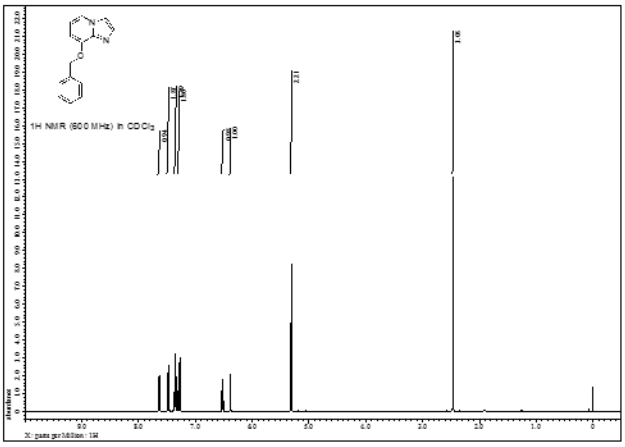

SCH-CN^{-}

^{1}H NMR ($CDCl_3$, 600 MHz) δ
7.64 (d, *J* = 6.9 Hz, 1H),
7.48 (d, *J* = 7.6 Hz, 1H),
7.35 (t, *J* = 7.6 Hz, 2H),
7.30 (t, *J* = 7.6 Hz, 1H),
7.29 (s, 1H),
6.52 (dd, *J* = 7.6, 6.9 Hz, 1H),
6.38 (d, *J* = 7.6 Hz, 1H),
5.31 (s, 2H),
2.46 (s, 3H).

Fig. S6 ^{1}H NMR spectrum of SCH28080 and its derivatives synthesised in this study.

Two-dimensional crystal	
Space group	$p22_12_1$
Lattice constants	a = 142.6 Å, b = 112.0 Å, c = 320.0 Å (assumed), γ = 90.0°
Electron micrographs	
Approximate tilt angle	No. of images
0°	9
20°	21
45°	101
60°	147
Total	278
Resolution limit	
Parallel to the membrane plane	6.5 Å
Perpendicular to the membrane plane	7.6 Å
Maximum tilt angle	64.0°
Range of underfocus	4,200 ~ 31,000 Å
Number of observed reflections	59,212 (8,440)[a]
unique reflections	4,974 (433)
Overall weighted phase residuals[b]	33.1° (52.9°)
Overall weighted R-factor[a]	0.469 (0.481)

Table S1 Electron crystallographic data

[a]Values in parentheses refer to data in the highest resolution shell (7.1 - 6.5 Å).

[b]90° is random. Used reflections are ≤ IQ 7.

position	mutation	$K_m(K^+)$, mM	V_{max}, μmol/mg/h	*n*
WT	-	1.4 ± 0.3	3.7 ± 1.8	16
D136	A	3.2 ± 0.5	1.7 ± 0.9	6
	L	2.3 ± 0.5	4.3 ± 1.5	6
	I	2.4 ± 0.1	2.5 ± 0.7	5
	F	2.9 ± 0.9	3.7 ± 0.8	5
D137	A	1.5 ± 0.3	2.6 ± 0.4	8
	L	1.4 ± 0.2	2.2 ± 0.8	6
	I	1.0 ± 0.2	1.7 ± 0.6	6
	F	1.0 ± 0.3	1.4 ± 0.4	6
N138	A	0.9 ± 0.2	3.0 ± 1.0	8
	L	0.9 ± 0.1	2.0 ± 0.3	6
	I	2.3 ± 0.5	1.3 ± 0.2	8
	F	0.6 ± 0.1	1.8 ± 0.3	6
L139	A	1.0 ±0.1	4.1 ±2.8	5
	-			
	I	1.6 ± 0.2	3.7 ± 0.8	5
	F	0.9 ± 0.1	4.1 ± 0.8	6
Y140	A	1.2 ± 0.3	3.0 ± 1.1	5
	L	0.9 ± 0.2	5.1± 1.9	5
	I	0.9 ± 0.2	4.6 ± 1.6	5
	F	1.1 ± 0.2	6.0 ± 1.8	5
L141	A	n.d.[a]	n.d.	3
	-			
	I	1.8 ± 0.5	1.2 ± 0.3	6
	F	n.d.	n.d.	3

Table S2 Kinetic parameters for M2 mutants

Michaelis constant for K^+ (K_m, mM) and maximum ATPase activity (V_{max}, μmol/mg/h)

determined by K^+-dependent ATPase activity measurement in the absence of inhibitor (Fig. S4) for evaluated mutants are indicated. Values represent mean ± SD from the indicated number (*n*) of the H^+,K^+-ATPase measurement using the membrane fraction obtained from at least three independent expression trials. [a]Not determined.

		K_i[SCH28080]		K_i[BYK99]	
TM	mutant	nM	-fold[a]	nM	-fold
-	WT	150 ± 10	1.0	5.8 ± 0.1	1.0
M4	V331A	1000 ± 100	6.7	47 ± 3	8.1
	V331F	210 ± 30	1.4	15 ± 2	2.3
	F332I	310 ± 40	2.1	8.9 ± 0.7	1.5
	M334F	100 ± 15	0.67	16 ± 4	2.8
	V338A	600 ± 40	4.0	50 ± 7	8.6
	A339S	320 ± 10	2.1	21 ± 1	3.6
M5	L796F	1300 ± 200	8.7	**106 ± 10**	**18**
	L800F	1000 ± 200	6.7	**59 ± 5**	**10**

Table S3 K_i values of the mutants that do not significantly affect the P-CAB binding.

Data are shown as in Table 1.
